# Zero coronary calcium in the presence of three-vessel and left main coronary artery disease in a Hodgkin lymphoma survivor

**DOI:** 10.1007/s12471-015-0719-0

**Published:** 2015-06-30

**Authors:** E.M. Engbers, M. Mouden, P.L. Jager, J.R. Timmer

**Affiliations:** 1 Department of Cardiology, Isala Hospital, Dokter Van Heesweg 2, 8025 AB Zwolle, The Netherlands; 2 Department of Nuclear Medicine, Isala Hospital, Zwolle, The Netherlands

**Keywords:** Coronary artery calcium, Radiation therapy

## Abstract

We describe a 45-year-old male survivor of Hodgkin lymphoma, treated with mediastinal radiation therapy, referred for single-photon emission computed tomography (SPECT) myocardial perfusion imaging in combination with coronary artery calcium (CAC) scoring. SPECT demonstrated a reversible moderate-sized lateral perfusion defect, and the CAC score was zero. A calcium score of zero markedly reduces the probability of having coronary artery disease (CAD) and is associated with a very low risk of future cardiovascular events. However, a CAC score of zero does not completely rule out obstructive CAD. In this case, invasive coronary angiography revealed three-vessel CAD with left main involvement. Whether mediastinal radiation therapy in general is associated with CAD without accompanying CAC is yet unclear.

## Introduction

Patients with prior supradiaphragmatic radiotherapy for Hodgkin lymphoma are at increased risk for premature coronary artery disease (CAD) [1–4]. Recently, it was suggested that coronary artery calcium (CAC) scoring may be used as a screening tool for CAD in this patient group [3]. However, it is currently unknown whether CAC scoring in this specific patient group has the same clinical value as in the general population.

A 45-year-old male survivor of Hodgkin lymphoma, who underwent mediastinal radiation therapy (involved field radiation, 40 Gy) 20 years earlier, presented in our outpatient cardiology department with stable exercise-induced burning chest pain. His risk profile consisted of mild hypercholesterolaemia for which a statin was prescribed. The pretest likelihood for presence of CAD was intermediate (69 %) [5]. The resting electrocardiogram (ECG) was normal and echocardiography showed normal left ventricular function without significant valve abnormalities.

The patient was referred for single photon emission computed tomography (SPECT) and CAC scoring as is standard practice in our department. Stress was performed using adenosine and images were acquired on a cadmium zinc telluride (CZT)-based gamma camera (Discovery NM/CT 570c, GE Healthcare). CAC scoring was performed on non-contrast enhanced computed tomography images, which were acquired ECG triggered at 75 % of the R-R interval and were analysed using Smartscore software (GE Healthcare). The following scanning parameters were applied: 40 sections and 2.5 mm section thickness; gantry rotation time 330 ms; tube voltage 120 kV; and a tube current of 125 mA. The quality of the CAC scoring images was excellent.

**Fig. 1:**
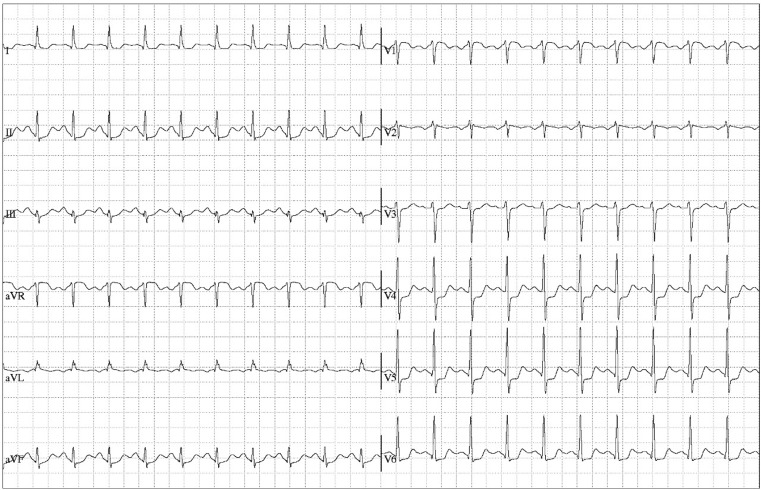
Electrocardiogram during adenosine infusion demonstrated ST-segment elevation in lead AVR and significant ST-segment depression in leads V4-V6 and lead II

**Fig. 2:**
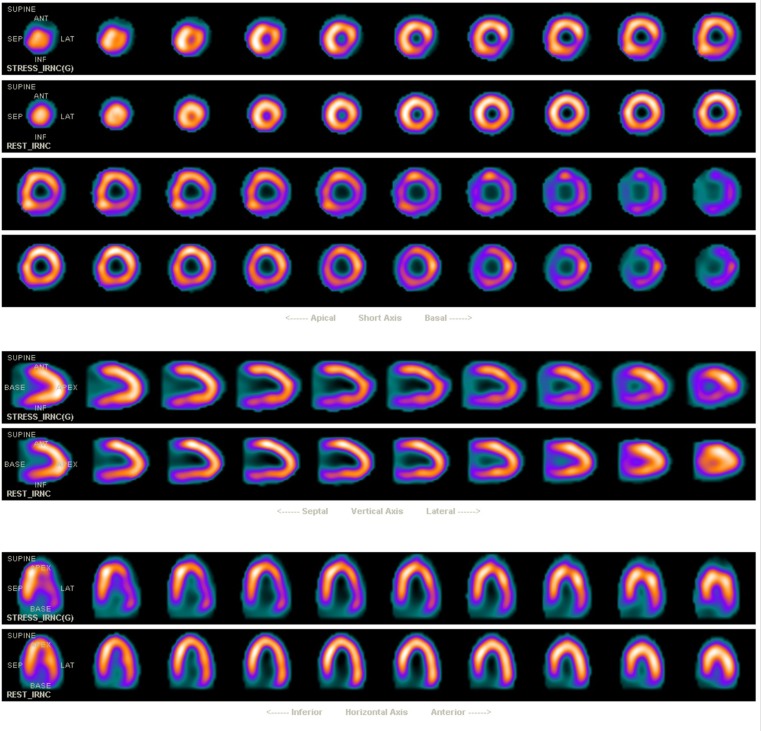
Myocardial perfusion single-photon emission computed tomography without attenuation correction. A reversible moderate size lateral perfusion defect is demonstrated

The ECG recordings during adenosine administration showed a heart rate increase from 100 to 131 beats per minute with extensive ST-segment deviation, suggestive of myocardial ischaemia (Fig. 1). CAC scoring did not detect any calcium deposits in the coronary tree, resulting in a CAC score of zero. SPECT showed a reversible moderate-sized lateral perfusion defect on both attenuation corrected and uncorrected images (Fig. 2). Gated post-stress images showed a mildly reduced ejection fraction (45 %) and an end-diastolic volume of 109 ml. No gated rest images were acquired.

**Fig. 3:**
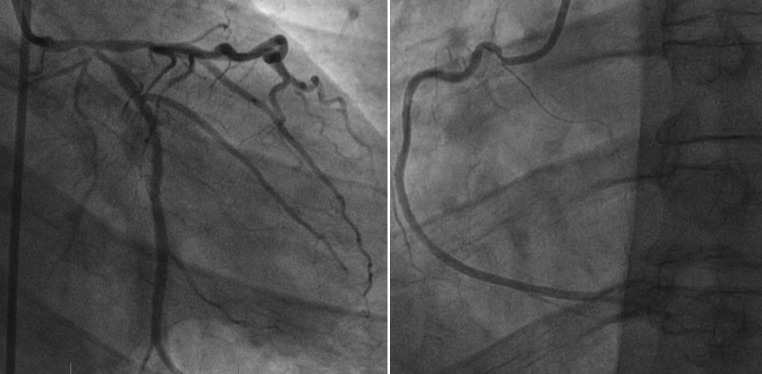
The left main demonstrated significant stenosis with involvement of the ostia of the left anterior descending and circumflex artery. The ostial right coronary artery demonstrated significant stenosis. No other stenoses were found in the course of the coronary arteries

Because of the scan results and persistent symptoms unresponsive to optimal medical therapy invasive coronary angiography was performed, which revealed relevant three-vessel disease with left main (LM) involvement (Fig. 3). Uncomplicated coronary artery bypass grafting was performed 4 days later, with grafting of the left internal mammary artery to left anterior descending artery and radial artery to diagonal artery to posterolateral branch to posterior descending artery.

## Discussion

A calcium score of zero markedly reduces the probability of having CAD. Also, there is ample evidence confirming very low risk of future cardiovascular events in a symptomatic population with zero CAC [6]. Hence, the absence of coronary calcium has been proposed as a selection tool for invasive coronary angiography [7]. However, a calcium score of zero does not completely rule out obstructive CAD. Two studies [8, 9] which performed coronary computed tomography angiography (CTA) in symptomatic patients found obstructive CAD (≥ 50 % stenosis) in approximately 4 % of the patients with a CAC score of zero. Moreover, both studies [8, 9] demonstrated that this non-calcified obstructive CAD was associated with increased cardiovascular events. It must be stressed that the prevalence of significant CAD in the absence of coronary calcium largely depends on the population which is studied. Factors predisposing to a higher risk of significant CAD despite absent coronary calcium are relatively young age, high pretest likelihood of the disease and typical angina [6, 10]. These characteristics are similar to the case presented here, except the pretest likelihood in this patient was considered intermediate [5]. However, one must realise that this crude standardised risk assessment does not include patient specific characteristics, such as previous supradiaphragmatic radiotherapy.

Whether patients treated with mediastinal radiotherapy are also at increased risk of CAD despite the absence of coronary calcium is unknown. Differences in plaque morphology between radiation-induced atherosclerosis and ‘traditional’ atherosclerosis were described by Stewart et al. [11], who found radiation-induced plaques to be more commonly inflammatory and more prone to intraplaque haemorrhage. However, it is not known whether this results in more non-calcified plaques in radiation-induced atherosclerosis.

Studies investigating CAD with CAC scoring or coronary CTA in survivors of Hodgkin lymphoma are scarce but demonstrate that subclinical CAD is common [12, 13]. In a study by Rademaker et al. [12], six out of nine patients had CAC scores above the 90th percentile for their age and sex group and the patient without CAD had a CAC of zero. This would suggest that premature CAD in this population is associated with an increase in coronary calcium, similar to CAD in the general population.

The extent of ischaemia in this case report is probably underestimated by SPECT. This is a well-known limitation of myocardial perfusion imaging in patients with left main stenosis or obstructive 3-vessel disease [14], because it determines relative perfusion. Thereby, it relies on the normalisation of acquired data to the region of the myocardium with maximal perfusion, even if this area of perfusion may not be normal.

In summary, although there is no evidence suggesting a greater risk of non-calcified plaques in patients treated with mediastinal radiotherapy, the current case report shows that we should interpret the absence of CAC with caution in this specific patient group. Typical cardiac chest pain should not be analysed solely by CAC scoring, as a CAC score of zero cannot exclude a high-risk coronary anatomy and therefore additional coronary CTA should be considered.

### Funding

None.

### Conflict of interest

None declared.

## References

[1] Reinders JG, Heijmen BJ, Olofsen-van Acht MJ, van Putten WL, Levendag PC (1999). Ischemic heart disease after mantlefield irradiation for Hodgkin’s disease in long-term follow-up. Radiother Oncol.

[2] Swerdlow AJ, Higgins CD, Smith P (2007). Myocardial infarction mortality risk after treatment for Hodgkin disease: a collaborative British cohort study. J Natl Cancer Inst.

[3] van Leeuwen-Segarceanu EM, Bos WJ, Dorresteijn LD (2011). Screening Hodgkin lymphoma survivors for radiotherapy induced cardiovascular disease. Cancer Treat Rev.

[4] Aleman BM, van den Belt-Dusebout AW, De Bruin ML (2007). Late cardiotoxicity after treatment for Hodgkin lymphoma. Blood.

[5] Genders TS, Steyerberg EW, Alkadhi H (2011). A clinical prediction rule for the diagnosis of coronary artery disease: validation, updating, and extension. Eur Heart J.

[6] Sarwar A, Shaw LJ, Shapiro MD (2009). Diagnostic and prognostic value of absence of coronary artery calcification. JACC Cardiovasc Imaging.

[7] Greenland P, Bonow RO, Brundage BH (2007). ACCF/AHA 2007 clinical expert consensus document on coronary artery calcium scoring by computed tomography in global cardiovascular risk assessment and in evaluation of patients with chest pain: a report of the American College of Cardiology Foundation Clinical Expert Consensus Task Force (ACCF/AHA Writing Committee to Update the 2000 Expert Consensus Document on Electron Beam Computed Tomography) developed in collaboration with the Society of Atherosclerosis Imaging and Prevention and the Society of Cardiovascular Computed Tomography. J Am Coll Cardiol.

[8] Kim YJ, Hur J, Lee HJ (2012). Meaning of zero coronary calcium score in symptomatic patients referred for coronary computed tomographic angiography. Eur Heart J Cardiovasc Imaging.

[9] Villines TC, Hulten EA, Shaw LJ (2011). Prevalence and severity of coronary artery disease and adverse events among symptomatic patients with coronary artery calcification scores of zero undergoing coronary computed tomography angiography: results from the CONFIRM (Coronary CT Angiography Evaluation for Clinical Outcomes: An International Multicenter) registry. J Am Coll Cardiol.

[10] van Werkhoven JM, de Boer SM, Schuijf JD (2010). Impact of clinical presentation and pretest likelihood on the relation between calcium score and computed tomographic coronary angiography. Am J Cardiol.

[11] Stewart FA, Heeneman S, Te Poele J (2006). Ionizing radiation accelerates the development of atherosclerotic lesions in ApoE-/- mice and predisposes to an inflammatory plaque phenotype prone to hemorrhage. Am J Pathol.

[12] Rademaker J, Schöder H, Ariaratnam NS (2008). Coronary artery disease after radiation therapy for Hodgkin’s lymphoma: coronary CT angiography findings and calcium scores in nine asymptomatic patients. AJR Am J Roentgenol.

[13] Kupeli S, Hazirolan T, Varan A (2010). Evaluation of coronary artery disease by computed tomography angiography in patients treated for childhood Hodgkin’s lymphoma. J Clin Oncol.

[14] Berman DS, Kang X, Slomka PJ (2007). Underestimation of extent of ischemia by gated SPECT myocardial perfusion imaging in patients with left main coronary artery disease. J Nucl Cardiol.

